# Atom‐by‐Atom Multimetal CuMoP Deposition on Carbon Electrodes for Sensitive Antidiabetic Metformin Electrooxidation

**DOI:** 10.1002/ansa.70070

**Published:** 2026-02-27

**Authors:** Hong Wan, Mingfang Zhan, Chunyan Yin, Sima Akter, Sakil Mahmud

**Affiliations:** ^1^ School of Life Science Wuchang University of Technology Wuhan People's Republic of China; ^2^ Faculty of Medicine Enam Medical College and Hospital University of Dhaka Dhaka People's Republic of Bangladesh; ^3^ Department of Chemistry and Physics Ivory V. Nelson Center for the Sciences Lincoln University Oxford Pennsylvania USA; ^4^ Department of Textile Engineering Faculty of Engineering Daffodil International University Dhaka People's Republic of Bangladesh

**Keywords:** antidiabetic metformin, atom‐by‐atom deposition, Cu–Mo–P nanocomposite, electrochemical sensor, mixed‐valence catalysis, screen‐printed carbon electrode

## Abstract

An atom‐by‐atom ternary Cu–Mo–P nanocomposite was electrochemically co‐deposited onto screen‐printed carbon electrodes (SPCEs) to form a highly active sensing interface for the determination of metformin (MF). The one‐step anodic fabrication yielded a porous, uniformly distributed network of mixed‐valence Cu^+^/Cu^2+^ and Mo^6+^ centres embedded within a phosphate‐stabilized matrix. Comprehensive microstructural and spectroscopic analyses revealed enhanced surface roughness, expanded electroactive area and favourable charge‐transfer characteristics. The CuMoP/SPCE exhibited markedly amplified redox activity and proton‐coupled electron‐transfer behaviour toward MF, enabling sensitive detection with a linear range of 0.99–13.82 µM and a low detection limit of 0.85 µM. Excellent selectivity, stability and reproducibility were achieved, with recoveries of 94.32%–107.23% in pharmaceutical samples and negligible interference from common ions or saccharides. These results demonstrate the synergistic catalytic contributions of Cu, Mo and P in enabling efficient electron mediation, positioning the CuMoP/SPCE robust, low‐cost platform for rapid pharmaceutical quality control and therapeutic monitoring.

## Graphical abstract

An atom‐by‐atom ternary Cu–Mo–P nanocomposite is electrochemically co‐deposited onto screen‐printed carbon electrodes to create a highly active sensing interface for metformin detection. The porous mixed‐valence network enhances electroactive surface area and charge transfer, enabling sensitive and selective electrooxidation with a low detection limit. The platform demonstrates reliable performance in pharmaceutical samples for rapid therapeutic monitoring.

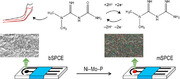

## 1 Introduction

Metformin (MF) (*N*,*N*‐dimethylbiguanide) remains the first‐line therapeutic agent for Type II diabetes mellitus and is one of the most widely prescribed small‐molecule drugs globally [1]. Its clinical utility extends beyond glycaemic control, with emerging evidence indicating roles in cardioprotection, cancer metabolism and ageing biology [2, 3]. The increasing prevalence of diabetes and the corresponding rise in MF consumption have generated pressing analytical challenges: accurate therapeutic monitoring, counterfeit‐drug screening and environmental surveillance of pharmaceutical residues [4, 5]. Conventional quantification strategies (such as chromatography [6], mass spectrometry [7], capillary electrophoresis [8] and spectrophotometry [9]) offer excellent accuracy but are hindered by high operational costs, labour‐intensive sample preparation and the need for specialized infrastructure [10]. These limitations motivate the development of alternative sensing platforms that combine high analytical performance with portability, operational simplicity and low cost.

Electrochemical sensing has emerged as a powerful approach for pharmaceutical analysis, offering rapid signal transduction, compatibility with miniaturization and the ability to detect analytes at low concentrations through carefully engineered interfacial chemistries [11, 12]. Among available transducer materials, screen‐printed carbon electrodes (SPCEs) have become indispensable owing to their scalability, low fabrication cost and adaptability to point‐of‐care formats [13, 14]. SPCEs allow precise control over electrode geometry and surface chemistry, enabling reproducible electrochemical behaviour and mass deployment in disposable sensor formats [15]. Despite these advantages, the intrinsic performance of unmodified SPCEs is often limited by sluggish electron‐transfer kinetics, restricted electroactive surface area (ECSA) and the presence of nonconductive binders in carbon inks. As a result, surface modification strategies are essential to unlock their full sensing potential.

A broad spectrum of nanomaterials has been explored to enhance SPCE performance, including carbon nanostructures (graphene, CNTs) [16], noble metals (Au, Pt and Ag) [17], transition‐metal oxides [18] and polyoxometalate clusters [19]. These nanoengineered interfaces can introduce new redox centres, improve adsorption affinity, accelerate charge transport, and increase surface roughness. However, many modification strategies rely on multi‐step syntheses, high‐temperature processing or unstable dispersions, which hinder reproducibility and scalability. Thus, there is significant interest in developing simple, controllable and robust electrochemical methods to fabricate nanostructured interfaces directly on SPCE surfaces.

Transition‐metal multicomponent systems, particularly those incorporating synergistic interactions between two or more metals and heteroatoms, offer unique opportunities for catalytic enhancement. Copper (Cu)‐ and molybdenum (Mo)‐based materials are especially appealing: Cu species (Cu^+^/Cu^2+^) provide efficient redox mediation and strong catalytic activity toward nitrogen‐containing pharmaceuticals [20], whereas Mo‐oxides (Mo^6+^/Mo^5+^) exhibit strong electron‐withdrawing characteristics and high capacity for modulating surface charge density [21]. Incorporating phosphorus adds an axis of tunability by forming metal–phosphate frameworks that stabilize mixed‐valence states, improve interfacial adhesion and modulate proton‐coupled electron‐transfer pathways [22, 23]. Yet, the integration of Cu, Mo and P into a unified composite film through a single‐step, electrochemically driven process remains underexplored—despite the potential to create highly conductive, porous and catalytically rich interfacial architectures.

MF poses specific electrochemical detection challenges due to its highly hydrophilic nature, the absence of aromaticity and the electro‐inactive character on most conventional electrodes [5]. To achieve efficient oxidation, electrode surfaces must provide active sites that facilitate electron transfer to the electron‐rich biguanide moieties via adsorption‐mediated or catalytic pathways. Prior studies have typically relied on single‐item modifications, such as noble‐metal catalysts [24], metal oxides [25, 26, 27], polymeric films [28, 29] or carbon nanomaterials [16]. However, these approaches often exhibit limited stability, narrow linear ranges or low Faradaic efficiency. Moreover, few studies have leveraged ternary transition‐metal systems, despite their demonstrated advantages in electrocatalysis, including density‐of‐states manipulation, multiple redox channels and enhanced surface reactivity.

In this context, the electrochemical co‐deposition of a Cu–Mo–P composite onto SPCEs offers a compelling strategy for engineering multifunctional sensing interfaces. Electrochemical deposition provides precise control over film thickness, nucleation dynamics and oxidation state distribution, enabling the formation of uniform, adherent nanostructures without the need for surfactants or thermal treatments. Like Cu–Ni–Mo [30], the simultaneous incorporation of Cu and Mo species, stabilized by phosphate and hydroxide frameworks, may yield a hierarchical porous structure rich in redox‐active centres. Such architectures could enhance charge transfer, increase the ECSA and provide favourable binding environments for MF, ultimately improving analytical performance.

Here, we report the controlled electrochemical fabrication of a Cu–Mo–P‐modified SPCE (CuMoP/SPCE) for the sensitive and robust detection of MF. We combine comprehensive physicochemical characterization to elucidate the composition, morphology and electronic structure of the CuMoP interface. Electrochemical analyses demonstrate that CuMoP yields substantial enhancements in electron‐transfer kinetics, surface reactivity and catalytic response toward MF. The resulting sensor exhibits high sensitivity, low detection limits, broad linear range and strong stability, positioning CuMoP/SPCE as a promising platform for pharmaceutical quality control and therapeutic monitoring. This work presents a ternary Cu–Mo–P composite film fabricated in a single step via an electrochemical process, highlighting the potential of multimetal–phosphate networks to enable reliable and selective electrochemical sensing on disposable SPCE platforms.

## 2 Experiential Procedures

### 2.1 Materials

Copper(II) sulphate (CuSO_4_), sodium hypophosphite monohydrate (NaH_2_PO_4_·H_2_O), sodium molybdate dihydrate [Na_2_MoO_4_·2H_2_O], potassium ferricyanide [K_3_Fe(CN)_6_], potassium nitrate (KNO_3_), potassium chloride (KCl), potassium dihydrogen phosphate dihydrate (KH_2_PO_4_·2H_2_O), sodium hydroxide (NaOH) and disodium hydrogen phosphate dodecahydrate (Na_2_HPO_4_·12H_2_O) were obtained from Sinopharm Chemical Reagent Co. Ltd. (Shanghai, China). MF hydrochloride sustained‐release tablets (labelled 500 mg MF hydrochloride per tablet) were purchased from Sibangde Pharmaceutical Group Co. Ltd. (Shandong, China). All chemicals were of analytical grade and used as received, without further purification. Ultrapure water (resistivity ≥18.3 MΩ cm) was used to prepare all reagents and standard solutions. Stock solutions were freshly diluted to the required concentrations using 0.1 M phosphate‐buffered saline (PBS, pH 7.0), which also served as the supporting electrolyte during electrochemical analyses.

### 2.2 Fabrication of CuMoP‐Modified SPCE

Commercially available SPCEs (4.0 × 0.5 cm^2^) comprising a carbon working electrode (2 mm diameter), a carbon counter electrode, and an Ag/AgCl reference electrode were used as the sensing platform. Before modification, the electrodes were electrochemically pretreated in 0.1 M PBS (pH 5.0) by cyclic voltammetry (CV) between −0.5 and +1.5 V at 50 mV s^−1^ for 10 consecutive cycles to remove residual binders and improve surface wettability. The CuMoP layer was subsequently deposited through a controlled electrochemical co‐deposition process in an aqueous solution containing 1.0 mM CuSO_4_, 1.0 mM Na_2_MoO_4_·2H_2_O and 0.5 mM NaH_2_PO_4_·H_2_O. A constant potential of +1.0 V (vs. Ag/AgCl) was applied for 60 s using a CHI660E potentiostat (Shanghai, China). This applied potential was used solely for anodic electrodeposition of the Cu–Mo–P composite and does not correspond to the analytical oxidation potential of MF. During deposition, the simultaneous reduction and interfacial oxidation of Cu^2+^, MoO_4_^2−^ and PO_4_^3−^ species led to the formation of a thin Cu–Mo–P composite film firmly adhered to the carbon surface. After deposition, the electrodes were rinsed thoroughly with ultrapure water, air‐dried and stored in PBS (pH 7.0) until use. The resulting CuMoP‐modified electrode was denoted mSPCE, whereas the bare unmodified electrodes were denoted bSPCE.

### 2.3 Characterization and Electrochemical Evaluation

The surface architecture and compositional features of the electrodes were examined using field‐emission scanning electron microscopy (FESEM, Zeiss Sigma 500, Germany) coupled with an energy‐dispersive x‐ray spectroscopy (EDS) detector to map the spatial distribution of Cu, Mo, P and O. The nanoscale topography and roughness variations of the CuMoP coating were analysed by atomic force microscopy (AFM, Bruker Dimension Icon, USA) in tapping mode, providing quantitative surface parameters such as root mean square (RMS) roughness. The electronic structure and oxidation states of the surface elements were determined by x‐ray photoelectron spectroscopy (XPS, AXIS Supra, Kratos Analytical Ltd., UK) using a monochromatic Al *K*α radiation source (1486.6 eV).

Electrochemical measurements were conducted using a CHI660E electrochemical workstation. CV and differential pulse voltammetry (DPV) were performed in 0.1 M PBS (pH 5.0) containing 1.0 mM K_3_Fe(CN)_6_ and 0.1 M KCl as the redox probe. The CV experiments were recorded within the potential window of −0.5 to +0.5 V at a scan rate of 50 mV s^−1^, whereas DPV was measured using a pulse amplitude of 50 mV and a pulse width of 50 ms. All measurements were performed under ambient laboratory conditions (25°C ± 1°C). The pH‐dependent measurements (pH 4–9) were conducted in 0.1 M PBS adjusted to the desired pH values.

### 2.4 Preparation and Analysis of MF

MF hydrochloride sustained‐release tablets were finely ground, and an accurately weighed portion was dissolved in 50 mL of ultrapure water under ultrasonication for 20 min. The resulting solution was centrifuged to remove insoluble material, and the supernatant was transferred to a 100 mL volumetric flask and diluted to volume with 0.1 M PBS (pH 7.0). Electrochemical determination of MF was performed by DPV at the CuMoP/SPCE electrode under optimized conditions. The standard addition method was applied to evaluate recovery and validate analytical accuracy.

## 3 Results and Discussion

### 3.1 Physicochemical Characterizations of mSPCE

**FIGURE 1:**
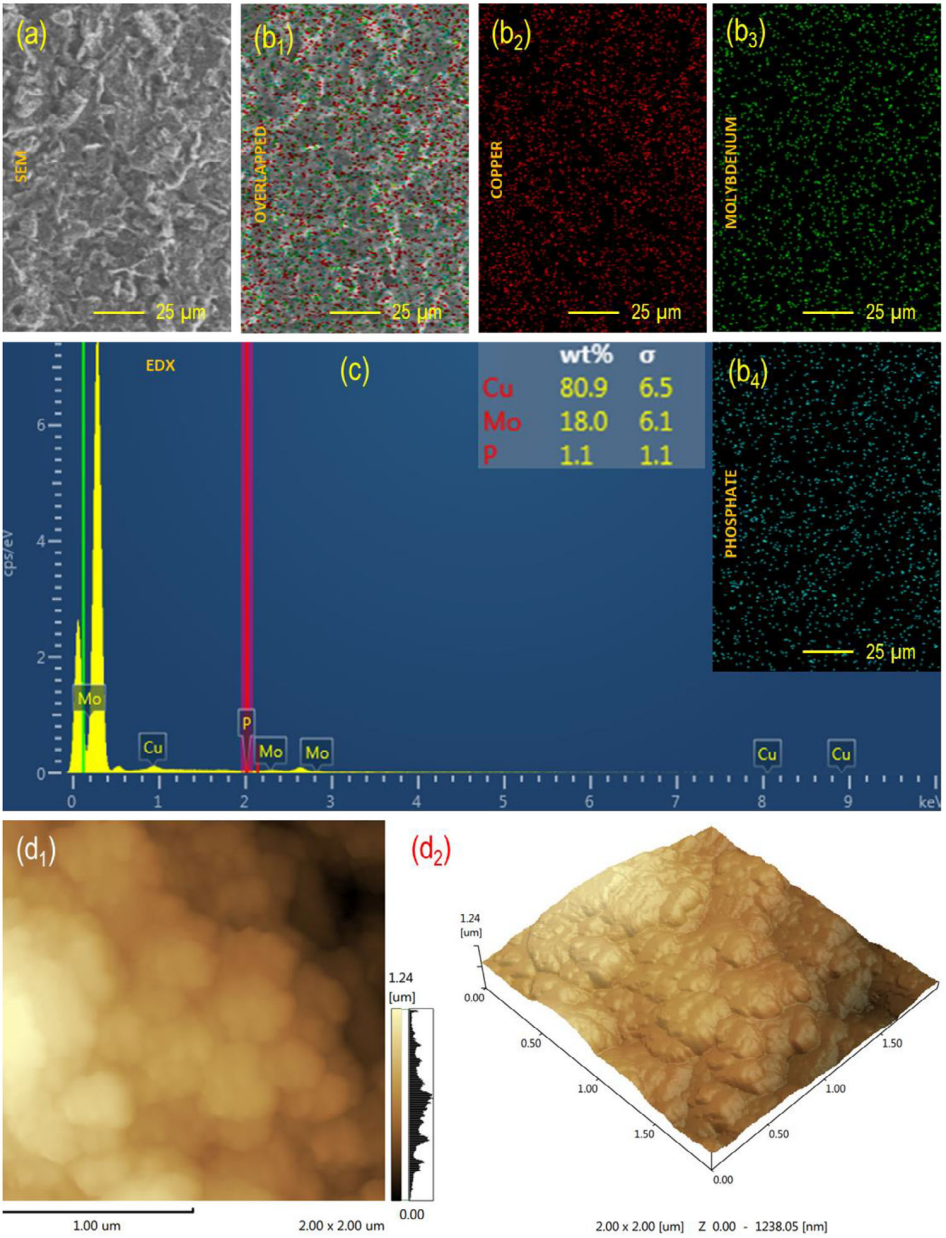
(a) SEM surface morphology, (b_1_–b_4_) elemental mapping, (c) EDS spectrum (with elemental composition inset), and (d_1_ and d_2_) AFM surface profile of the modified SPCE.

Morphological analysis by SEM revealed three distinct features: The bare SPCE displayed a smooth, featureless carbon layer; electrochemical activation introduced micro‐texturing and exposed edge‐like structures [31]; and CuMoP deposition produced a particulate, granular surface with a dense network of nanoscale aggregates and interstitial pores (Figure 1a), increasing surface area and providing abundant high‐curvature sites for heterogeneous charge transfer. Quantitative EDS of the CuMoP film showed a composition dominated by Cu (80.6 wt%), with appreciable Mo (18.0 wt%) and minor P (1.1 wt%), indicating surface enrichment of copper phases, incorporation of molybdenum, and a thin, phosphate‐bound layer acting as a structural binder (Figure 1c). Elemental mapping confirmed the co‐distribution of Cu and Mo across the substrate, with a continuous carbon background, indicating uniform lateral coverage rather than isolated islands (Figure 1b). This homogeneous, porous morphology enhances electrochemical performance by providing a high density of electroactive Cu/Mo centres, shorter diffusion paths and a larger true surface area. Collectively, SEM and EDS analyses confirm the successful, uniform formation of the CuMoP layer, and this explains the observed improvement in MF oxidation currents.

AFM of the mSPCE (Figure 1d), recorded over a 2.0 × 2.0 µm^2^ area, reveals a moderately rough and granular surface morphology, reflecting the physical topography of the CuMoP coating. The surface roughness parameters (*R*_a_ = 205.8 nm, *R*_q_ = 237.1 nm and *R*_z_ = 1.17 µm) indicate the formation of fine nanoclusters with well‐distributed protrusions and shallow valleys. This textured morphology reflects uniform deposition of CuMoP nanoparticles, effectively increasing the ECSA and improving electron transport pathways. Such nanoscale roughness enhances analyte accessibility and facilitates rapid charge transfer during the oxidation of analytes [32]. The balanced surface texture, with sufficient roughness and uniformity, confirms that the CuMoP layer forms a stable and catalytically active interface suitable for high‐performance electrochemical sensing.

To quantitatively assess the ECSA, CV of the Fe(CN)_6_^3−^/^4−^ redox couple was employed and analysed using the Randles–Ševčík equation (Equation 1). In this equation, $I_{p}$ is the peak current (*A*), n is the number of transferred electrons, A is the ECSA (cm^2^), D is the diffusion coefficient of Fe(CN)_6_^3−^/^4/−^ (7.6 × 10^−6^ cm^2^ s^−1^), C is the concentration (1.0 mM), and v is the scan rate (0.05 V s^−1^). The calculated ECSA values were 0.00121 cm^2^ for the bare SPCE and 0.00802 cm^2^ for the CuMoP/SPCE, corresponding to an approximately 6.7‐fold increase after modification. This confirms that the AFM‐observed roughness results in a significantly larger electroactive area, thereby amplifying the faradaic response.

(1) $$I_{p}=2.69\times 10^{5}n^{3/2}AD^{1/2}Cv^{1/2}$$

**FIGURE 2:**
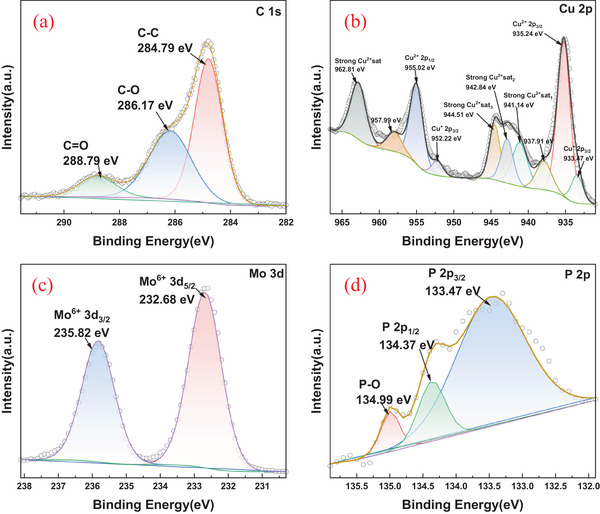
High‐resolution XPS of mSPCE: (a) C 1s, (b) Cu 2p, (c) Mo 3d and (d) P 2p regions.

The surface composition and chemical states of the mSPCE were examined using XPS (Figure 2). The spectrum confirmed the presence of Cu, Mo, P, O and C, validating the successful deposition of the CuMoP composite on the SPCE surface. The high‐resolution C 1s spectrum (Figure 2a) exhibited three distinct peaks at 288.79, 286.17 and 284.79 eV, assigned to C = O, C–O and C–C functional groups, respectively. The predominance of oxygen‐containing carbon species suggests the presence of partially oxidized carbon domains [33], which enhance surface polarity and favour metal–oxygen–carbon interactions, improving the electronic coupling between the CuMoP layer and the conductive carbon substrate. The Cu 2p spectrum revealed a complex pattern characterized by multiple peaks and pronounced shake‐up satellite features (Figure 2b). The principal peaks at 955.02 eV (Cu^2+^ 2p_1/__2_) and 935.24 eV (Cu^2+^ 2p_3/__2_), accompanied by strong satellites at 962.81, 957.99, 944.51, 942.84 and 941.14 eV, indicate the presence of Cu^2+^ species, typical of CuO or Cu(OH)_2_ [34]. In addition, peaks at 952.22 and 933.47 eV correspond to Cu^+^ 2p_1/__2_ and Cu^+^ 2p_3/__2_, respectively, confirming the coexistence of Cu^+^/Cu^2+^ redox states within the film. The dual‐valence nature of copper creates a dynamic electron‐transfer environment that enables redox cycling between Cu^+^ and Cu^2+^, which play a key role in facilitating the electrooxidation of aspirin by providing multiple active centres for charge mediation.

The Mo 3d region exhibited two well‐defined peaks at 235.82 eV (Mo^6+^ 3d_3/__2_) and 232.68 eV (Mo^6+^ 3d_5/__2_) (Figure 2c), indicative of Mo^6+^ oxidation states associated with molybdate or MoO_3_ species [35]. The high oxidation state of Mo implies its involvement in electron‐withdrawing processes that enhance charge delocalization and promote synergistic redox interactions with copper centres. The P 2p spectrum showed peaks at 134.99 eV (P–O), 134.37 eV (P 2p_1/__2_) and 133.47 eV (P 2p_3/__2_) (Figure 2d), characteristic of phosphate (PO_4_^3−^) groups [36]. The phosphate framework likely serves as a structural stabilizer, forming strong metal–oxygen–phosphate linkages that enhance the composite's overall durability and electrochemical stability [37]. Collectively, the XPS analysis confirms the formation of a mixed‐valence Cu–Mo–P oxide‐phosphate network on the SPCE surface. The coexistence of Cu^+^/Cu^2+^ and Mo^6+^ species, integrated within a phosphate matrix, establishes an electronically conductive and chemically stable environment that enhances both electron mobility and proton transfer during electrochemical reactions. These features underpin the superior electrocatalytic activity and long‐term operational stability of the mSPCE electrode for MF detection.

### 3.2 Electrochemical Behaviour of MF on mSPCE

**FIGURE 3:**
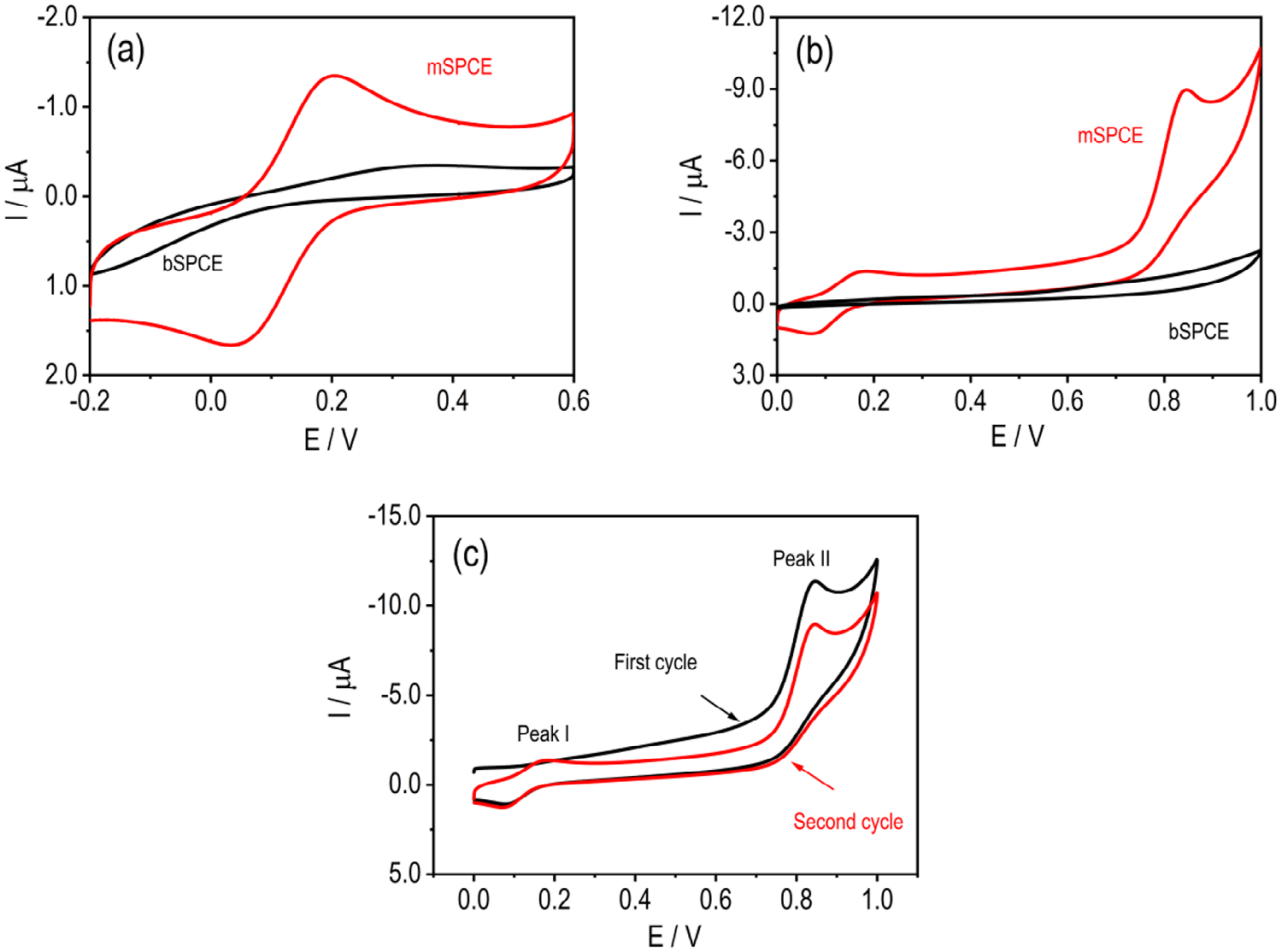
(a) CV recorded in a 1:1 mixture of K_3_[Fe(CN)_6_]/K_4_[Fe(CN)_6_] (1.0 mM each) in 0.1 M KCl at a scan rate of 20 mV s^−1^. (b) CV responses of MF (1 mM, PBS pH 5.0) at bSPCE and mSPCE at a scan rate of 50 mV s^−1^. (c) Consecutive CV cycles of MF (1 mM, PBS pH 5.0) at mSPCE recorded at 50 mV s^−1^.

The CuMoP modification markedly enhanced the electrochemical activity of the SPCE, as confirmed by CV in 0.05 mM K_4_Fe(CN)_6_/K_3_Fe(CN)_6_ (50 mV s^−1^) (Figure 3a). The mSPCE exhibited well‐defined redox peaks at +0.065 and +0.22 V, in sharp contrast to the nearly featureless response of the bare bSPCE. The anodic peak current of mSPCE (1.33 µA) was ∼6.5 times higher than that of bSPCE (0.20 µA), corresponding to a similar enhancement in ECSA. The observed peak‐to‐peak separation of 155 mV indicates quasi‐reversible electron‐transfer kinetics, reflecting a significantly reduced charge‐transfer resistance. This improvement arises from the formation of a nanostructured CuMoP layer that exposes abundant electroactive sites, enhances conductivity, and introduces synergistic Cu–Mo redox centres that facilitate d‐orbital‐mediated charge transport. Phosphorus incorporation further modulates surface electronic properties, optimizing the interfacial energy landscape for rapid electron exchange. It is noted that electrochemical pretreatment alone can substantially improve the reversibility of the ferri/ferrocyanide redox couple on bare SPCEs. In this context, the response in Figure 3a is not intended to demonstrate superior intrinsic reversibility but rather to illustrate the pronounced current amplification and electroactive surface enrichment introduced by the CuMoP film. The quasi‐reversible behaviour and elevated capacitive contribution reflect the nanostructured, redox‐active nature of the composite layer rather than minimized interfacial resistance. These synergistic structural and electronic effects collectively enable efficient charge transfer and establish CuMoP as a robust electrocatalytic coating for high‐performance sensing platforms. The electrocatalytic advantage of CuMoP is therefore more appropriately assessed from its pronounced enhancement of MF oxidation currents relative to pretreated SPCEs, rather than from the ferri/ferrocyanide benchmark alone.

*The electrocatalytic comparison* between mSPCE and bSPCE for 1.0 mM MF in PBS (pH 5.0) showed a striking enhancement upon CuMoP modification (Figure 3b). Although bSPCE showed negligible oxidation signals, mSPCE produced two low‐potential redox peaks at −0.081 V (*E*_red_) and 0.16 V (*E*_ox_), accompanied by a strong anodic response at 0.85 V. The anodic and cathodic currents increased nearly tenfold, confirming that CuMoP substantially accelerates the interfacial kinetics and increases electroactive surface density. This improvement stems from the hierarchical, porous CuMoP network that provides a conductive scaffold decorated with redox‐active Cu and Mo sites. These centres promote electron tunnelling and proton‐coupled electron transfer (PCET), whereas phosphorus modulates local charge density and adsorption behaviour, enhancing MF–surface interactions. The low overpotential and well‐resolved redox features indicate efficient catalytic mediation and strong analyte binding to the modified surface. Collectively, the CuMoP nanocomposite transforms the inert SPCE into a highly sensitive and kinetically favourable interface for MF oxidation, characterized by amplified Faradaic currents, excellent signal definition and reproducible redox behaviour, attributes critical for analytical sensing applications.

*To elucidate interfacial redox dynamics*, the behaviour of 1.0 mM MF on mSPCE was monitored over successive potential cycles in PBS (pH 5.0) (Figure 3c). In the initial scan, a distinct reduction peak at −0.081 V and a dominant oxidation signal at 0.85 V were observed, suggesting an irreversible electron transfer involving the primary oxidation of MF on the fresh CuMoP surface. During the second cycle, the appearance of an anodic peak at 0.16 V coupled with the re‐emergence of −0.081 V established a quasi‐reversible redox pair, indicating the in situ formation of stable, surface‐confined intermediates. This transformation from irreversible to quasi‐reversible behaviour underscores the role of CuMoP in stabilizing PCET processes through its mixed‐valence Cu/Mo centres and electronically tuned P sites. The enhanced reversibility and reduced overpotential observed in subsequent cycles confirm both the catalytic robustness and structural integrity of the modified electrode. Such behaviour evidences efficient electron mediation and durable electrochemical activity, demonstrating that CuMoP not only facilitates rapid charge transfer but also preserves interfacial stability, key attributes for high‐performance, reproducible MF sensing.

**FIGURE 4:**
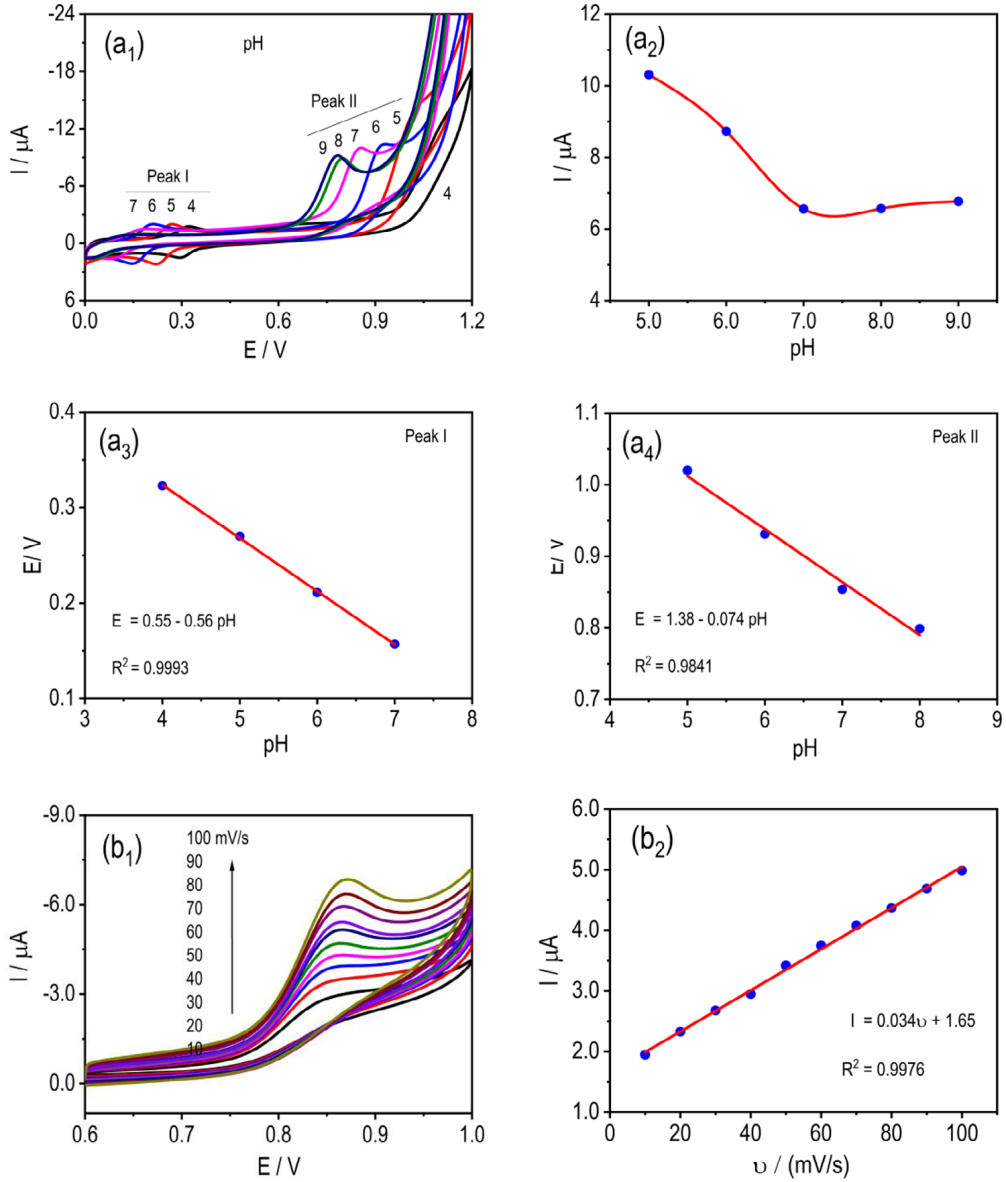
(a_1_–a_4_) CV responses of MF (1.0 mM) at the mSPCE recorded in 0.1 M PBS at different pH values (4–9) at 50 mV s^−1^. (b_1_ and b_2_) Scan‐rate‐dependent CVs of MF (1.0 mM) obtained at the mSPCE in pH 5.0 PBS and the corresponding linear relationship between anodic peak current and scan rate.

*The* pH*‐dependent electrochemical behaviour* of MF was evaluated in 0.1 M PBS with pH values ranging from 4 to 9 at a scan rate of 50 mV s^−1^ (Figure 4a_1_–a_4_). For the first redox process, the peak potential (*E*_1_) shifted negatively with increasing pH, following the linear regression *E*_1_ (*V*) = 0.55–0.056 pH (*R* ^2^ = 0.9993), whereas the second oxidation peak (*E*_2_) obeyed *E*_2_ (*V*) = 1.38–0.074 pH (*R* ^2^ = 0.9841). The negative shift in oxidation potential with increasing pH indicates a proton‐coupled electron‐transfer process, whereas the decrease in peak current at higher pH is attributed to reduced proton availability and partial deprotonation of surface‐active Cu/Mo sites, which weakens MF adsorption and catalytic efficiency. These negative shifts with increasing alkalinity confirm the direct involvement of protons in the electrode reaction, consistent with a proton‐coupled electron‐transfer mechanism. The oxidation peak current showed a strong pH dependence, peaking at pH 5.0 (10.31 µA) and decreasing steadily at higher pH values, indicating optimal catalytic activity under mildly acidic conditions. This trend suggests that both the protonation state of MF and the surface charge of CuMoP critically influence electron‐transfer efficiency. At higher pH, reduced proton availability and possible deprotonation of active sites diminish redox activity. Thus, pH 5.0 was identified as the optimal condition, balancing charge‐transfer kinetics and analyte adsorption for sensitive and stable MF oxidation on mSPCE.

*The effect of scan rate* on the electrochemical oxidation of MF (1.0 mM) at the mSPCE was examined in 0.1 M PBS (pH 5.0) over the range of 10–100 mV s^−1^ to elucidate the kinetic nature of the redox process (Figure 4b_1_–b_2_). A consistent increase in peak current with scan rate was observed, yielding a linear relationship described by *I* (µA) = 0.034*v* + 1.65 (*R* ^2^ = 0.9976). The linear dependence of peak current on scan rate within the investigated range suggests a significant contribution from adsorption‐controlled processes under these experimental conditions, whereas diffusion contributions cannot be completely excluded for a solution‐phase analyte. It indicates active participation of surface‐confined MF species in the charge‐transfer process. In addition, the peak current rises with scan rate due to faster electrochemical kinetics, whereas slower rates allow prolonged interaction, producing lower currents. The minimal shift in the oxidation peak potential with increasing scan rate indicates fast interfacial kinetics and efficient electron‐transfer mediation through the CuMoP layer. Such behaviour highlights the composite's excellent electrical conductivity and catalytic responsiveness, enabling rapid charge propagation without significant diffusion limitations. The dependence of current on scan rate thus confirms the quasi‐reversible nature of MF oxidation and validates the strong coupling between the analyte and the modified electrode surface. These results establish that CuMoP exhibits high interfacial reactivity and kinetic favourability, which are essential for achieving sensitive, stable and rapid electrochemical sensing of MF. A broader scan‐rate investigation would be required to fully deconvolute adsorption‐ and diffusion‐controlled contributions; however, the present data indicate that surface‐confined interactions play a dominant role in the observed electrocatalytic response.

The electrochemical modification process for fabricating the CuMoP/SPCE involves a controlled anodic electrodeposition at +1.0 V, leading to the synergistic co‐deposition of a Cu–Mo–P composite film. The mechanism begins with the electrochemical oxidation of hypophosphite, which generates reactive phosphate species and a localized alkaline microenvironment at the electrode surface (Equation I). This is complemented by its chemical dissociation, further increasing pH and producing phosphite (Equation II). The elevated pH triggers the precipitation of copper hydroxide (Equation III) and the condensation of molybdate to form molybdenum oxide (Equation IV). These metal hydroxides and oxides subsequently react with the generated phosphate/phosphite species, integrating into a cohesive, mixed‐valence oxide‐phosphate network that strongly adheres to the carbon surface (Equation V).

(I) $$H_{2}{\mathrm{PO}}_{2}^{-}+H_{2}O\to H_{2}{\mathrm{PO}}_{3}^{-}+2H^{+}+2e^{-}$$

(II) $$H_{2}{\mathrm{PO}}_{2}^{-}+OH^{-}\to {\mathrm{HPO}}_{3}^{2-}+1/2H_{2}$$

(III)

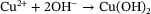

(IV) $${\mathrm{MoO}}_{4}^{2-}+2H^{+}\to \mathrm{Mo}O_{3}+H_{2}O$$

(V)

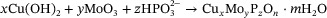




### 3.3 Analytical Performance of mSCPE

#### 3.3.1 Detection of MF

**FIGURE 5:**
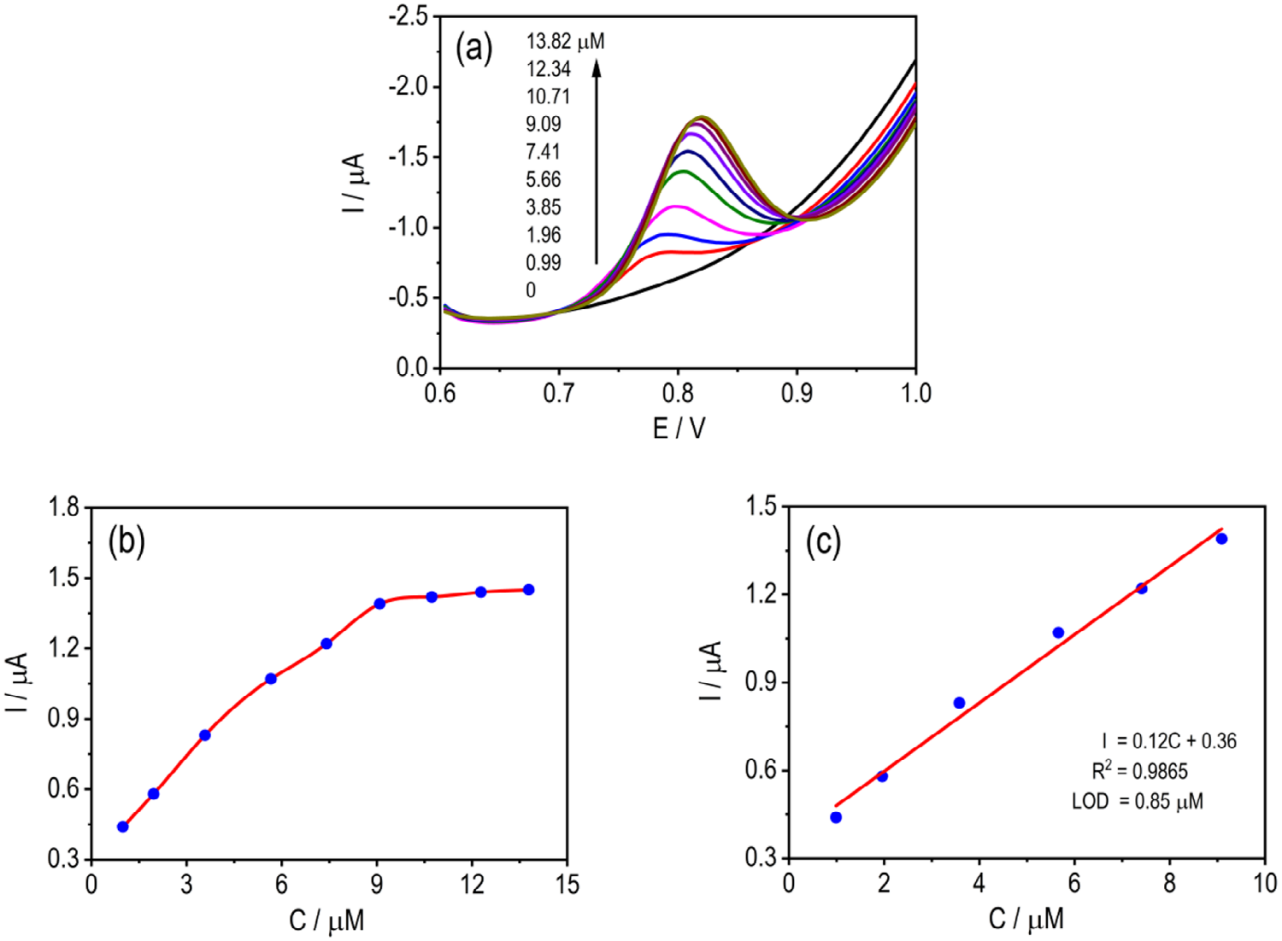
DPVs of mSPCE at different MF concentrations (a) and associated calibration curves (b and c) corresponding calibration plots obtained in PBS (pH 5.0) at a scan rate of 50 mV s^−1^.

The electrochemical detection of MF at the mSPCE was investigated using DPV in 0.1 M PBS (pH 5.0) at a scan rate of 50 mV s^−1^ (Figure 5a–c). The oxidation peak current increased progressively with increasing MF concentrations, and the peaks became sharper and more defined, accompanied by a slight positive potential shift. Although the oxidation peak of MF appears at ∼0.76 V versus Ag/AgCl, this potential lies within the operating window of numerous reported Cu‐, Co‐, and Ru‐based sensors (Table 1). Importantly, no significant current contributions from common inorganic ions or saccharides were observed even at large excesses, indicating that the CuMoP surface preferentially catalyses the oxidation of MF rather than nonspecific anodic responses. A strong linear correlation between current (*I*, µA) and concentration (*C*, µM) was observed: *I* = 0.12 *C* + 0.36 (*R* ^2^ = 0.9865), confirming the excellent sensitivity and reliability of the response. The limit of detection (LOD) and limit of quantification (LOQ) were determined from the calibration slope (*S* = 0.12 µA µM^−1^) using the standard analytical equations: LOD = 3*σ*/*S* and LOQ = 10*σ*/*S*, where *σ* represents the standard deviation of the blank response. On the basis of the experimental data, the LOD and LOQ were calculated as 0.85 and 2.83 µM, respectively, with a linear range of 0.99–13.82 µM. The steady rise in peak intensity and improved signal definition reflect enhanced electron‐transfer kinetics and strong adsorption affinity of MF on the CuMoP surface. The results are comparable with recent studies (Table 1). These findings confirm that mSPCE provides a robust, sensitive and low‐noise sensing interface suitable for trace‐level determination of MF with high analytical precision.

**TABLE 1:** Overview of electrochemical methods and their practical applications for MF detection [38].

Electrode	Method	Potential (**V**)	LOD (µM)	Linear range (µM)	Sample	Recovery (%)	Ref.
CuMoP/SPCE (mSPCE)	DPV	0.76	0.85	0.99–13.82	Tablet	94.32–107.23	This work
CB‐RuO_2_‐Nafion/GCE	DPV	1.12	0.70	10–70	Pharmaceutical products, river water, wastewater	101–110	[39]
CuMW/MWCNT/PE	CV	0.97	0.65	0.9–50	Tablet	98.2–103.5	[40]
*n*‐NC/CPE	AMP	0.66	0.45	4–63	Tablet, serum, urine, breast milk	97.1–109.4	[41]
CuBTC‐Nafion/GCE	DPV	1.07	0.19	0.5–280	Tablet, serum, urine	96.2–108.7	[42]
Co‐YIG/GCE	DPV	0.85	0.04	0–60	Serum	83.6–95	[43]
Cu‐BTC‐CNTs/GCE	CV	0.59	0.12	0.5–25	Pharmaceuticals	—	[44]
Cu‐GR/CPE	DPV	0.74	3.40	10.4–1125	Tablet, human serum	90.0–115.0	[45]
Cu(OH)_2_/CILE	SWV	0.6	0.50	1–4000	Tablet, human urine	96.6–106.4	[46]

CB–RuO_2_‐Nafion/GCE: carbon black–ruthenium oxide–Nafion modified glassy carbon electrode, Cu–GR/CPE: copper–graphene modified carbon paste electrode, CuBTC–Nafion/GCE: copper‐benzene‐1,3,5‐tricarboxylate–Nafion modified glassy carbon electrode, Co‐YIG/GCE: cobalt‐doped yttrium iron garnet modified glassy carbon electrode, CuMW/MWCNT/PE: copper molecular wire–multi‐walled carbon nanotube modified paste electrode, Cu‐BTC‐CNTs/GCE: copper‐benzene‐1,3,5‐tricarboxylate–carbon nanotubes modified glassy carbon electrode, Cu(OH)_2_/CILE: copper hydroxide modified carbon ionic liquid electrode, and n‐NC/CPE: nickel oxide nanotubes–carbon microparticles/Nafion nanocomposite modified carbon paste electrode.

Abbreviations: CV, cyclic voltammetry; DPV, differential pulse voltammetry; SPCE, screen‐printed carbon electrode.

**FIGURE 6:**
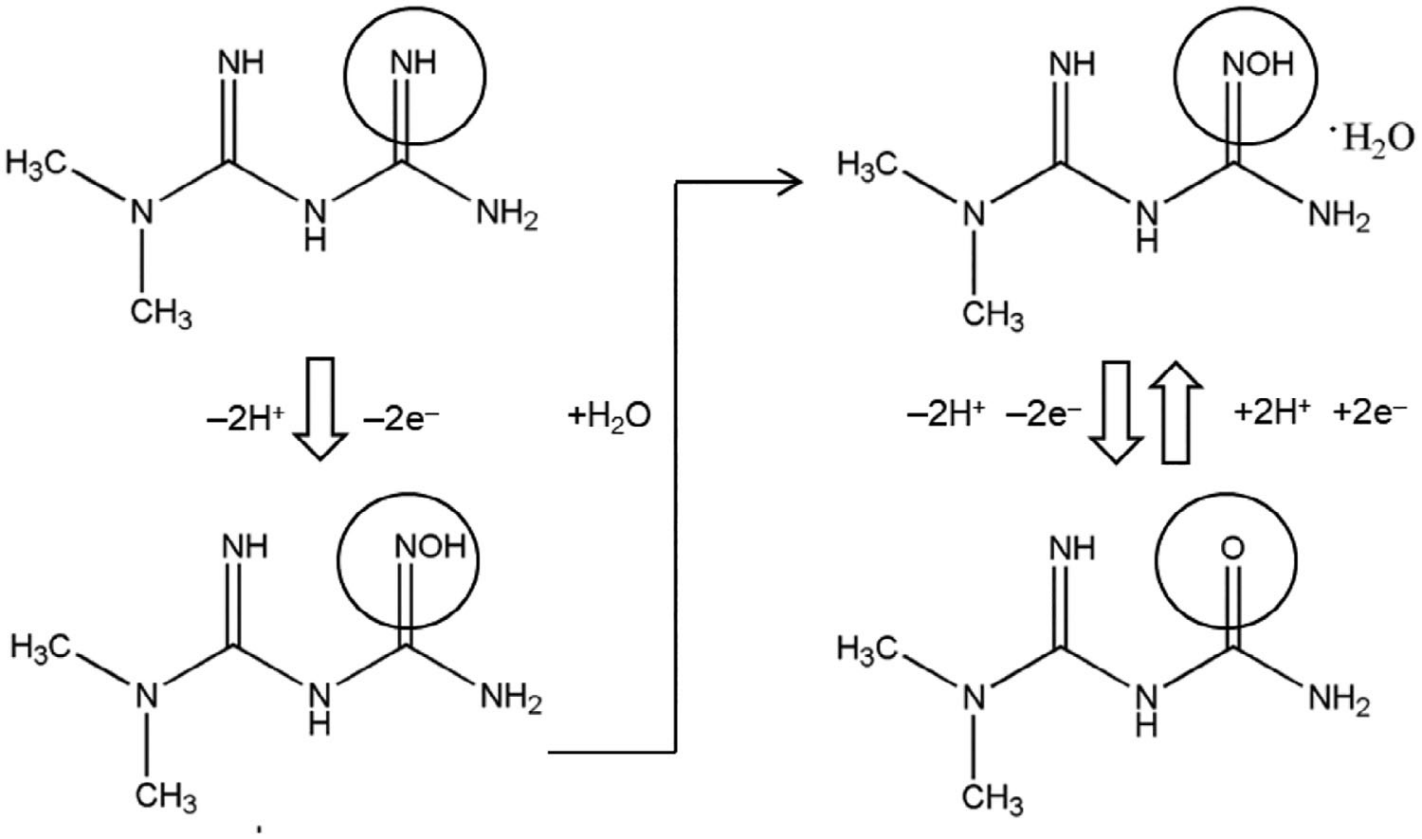
Electrochemical oxidation mechanism of MF on the mSPCE electrode.

The electrochemical detection of MF at the mSPCE follows a synergistic redox mechanism mediated by the mixed‐valence Cu–Mo–P oxide–phosphate framework. During electrode activation, the Cu^2+^/Cu^+^ and Mo^6+^/Mo^5+^ couples undergo dynamic redox transitions, enabling efficient electron and proton exchange across the composite. In a mildly acidic medium (pH 5.0), MF oxidation proceeds via a PCET pathway, facilitated by these redox‐active centres. The process begins with surface activation of copper and molybdenum species (Equation VI), generating catalytically active Cu(I) and Mo(V)–OH sites. The activated Cu(I)/Cu(II) pair then mediates electron transfer from MF, forming a radical cation intermediate (Equation VII). This intermediate undergoes hydrolytic conversion to a hydroxylated derivative (Equation VIII), followed by oxidative dehydrogenation yielding cyanoguanidine (CNG) as the final stable product (Equation IX). Throughout this process, Mo centres stabilize high oxidation states and facilitate charge delocalization, whereas phosphate groups maintain structural integrity and proton mobility. The overall sequence demonstrates that Cu, Mo and P act cooperatively to accelerate charge transport, lower the oxidation overpotential and enhance the electrocatalytic sensitivity of mSPCE for MF detection. The electrochemical oxidation mechanism of MF on the mSPCE electrode is schematically presented in Figure 6.

(VI) $$\mathrm{Cu}(\mathrm{II})-O-\mathrm{Mo}(\mathrm{VI})+H^{+}+e^{-}⇌\mathrm{Cu}(I)+\mathrm{Mo}(V)-\mathrm{OH}$$

(VII) $$\mathrm{MF}+\mathrm{Cu}(\mathrm{II})\to MF^{•+}+\mathrm{Cu}(I)$$

(VIII) $$MF^{•+}+H_{2}O\to \mathrm{HO}-\mathrm{MF}+H^{+}$$

(IX) $$\mathrm{HO}-\mathrm{MF}\to \mathrm{CNG}+2H^{+}+2e^{-}$$

#### 3.3.2 Real Sample Analysis

**FIGURE 7:**
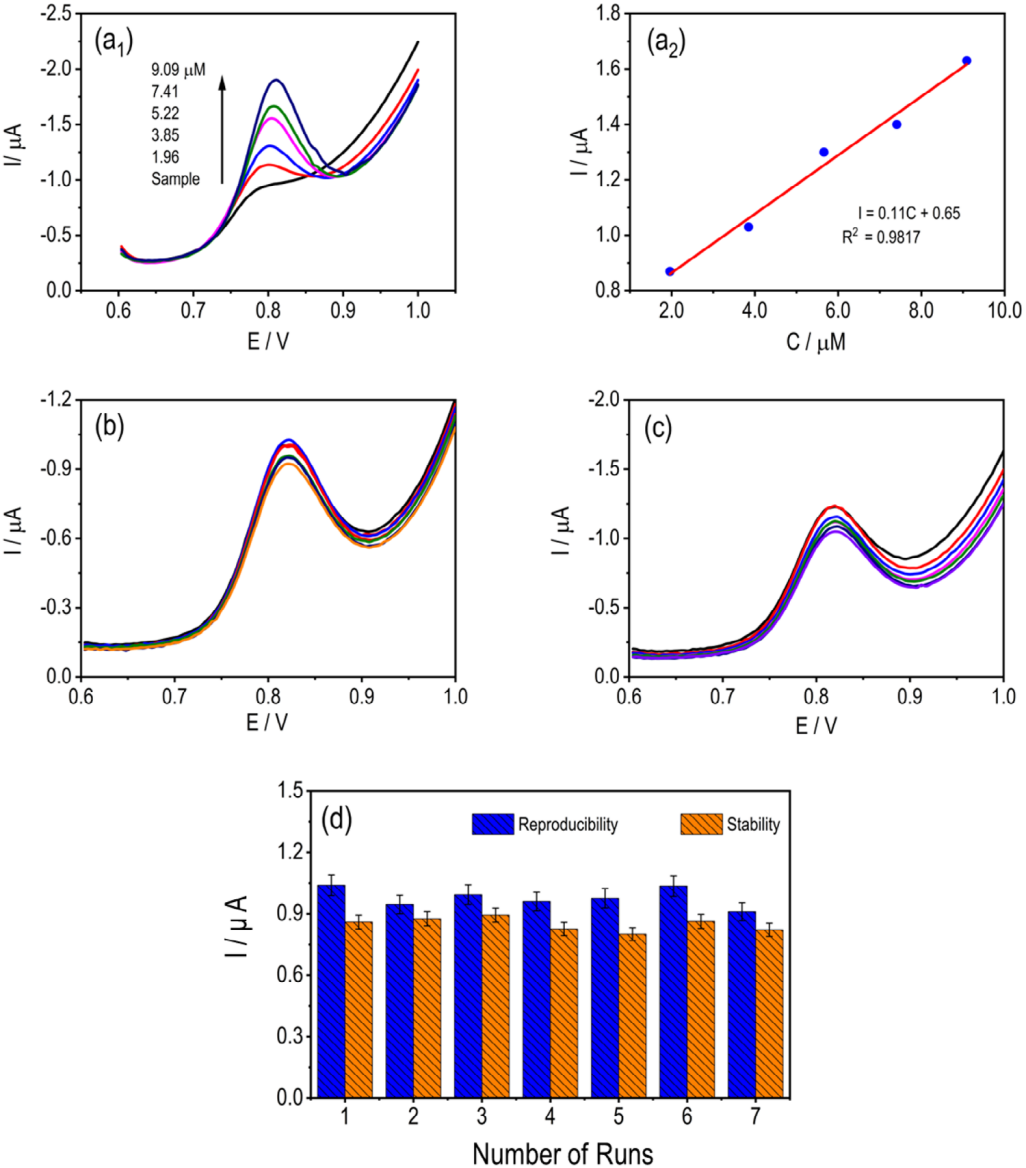
(a_1_ and a_2_) DPVs of MF on mSPCE recorded using the standard addition approach in PBS (pH 5.0) at 50 mV s^−1^, along with the corresponding calibration plot. (b–d) Evaluation of mSPCE stability and reproducibility: seven consecutive measurements on a single electrode and measurements across seven independently prepared electrodes, all in PBS (pH 5.0) containing MF at 50 mV s^−1^.

To verify the analytical accuracy and matrix independence of the mSPCE sensor, the standard addition method was employed in 0.1 M PBS (pH 5.0) at a scan rate of 50 mV s^−1^ (Figure 7a_1_–a_2_). An initial DPV scan of the unspiked sample exhibited no observable peak, confirming the absence of endogenous electroactive interference. Sequential additions of the MF standard produced progressively enhanced anodic responses, accompanied by minor positive potential shifts. The corresponding peak currents increased linearly with concentration, yielding the regression equation *I* (µA) = 0.11 *C* (µM) + 0.65 with a correlation coefficient of *R* ^2^ = 0.9817, indicating excellent proportionality between oxidation current and analyte level. This linearity confirms that the oxidation of MF at mSPCE is surface‐controlled and kinetically stable across the studied range [47]. The nearly parallel calibration slope to that obtained from the external standard method further validates the electrode's quantitative reliability. These results demonstrate that CuMoP modification provides a consistent, reproducible response even in the presence of sample matrix components, ensuring its applicability for direct determination of MF in pharmaceutical and biological formulations.

#### 3.3.3 Recovery Analysis

To further assess the accuracy and practical applicability of the mSPCE sensor, recovery experiments were conducted using MF tablet samples using the standard‐addition approach (*n* = 5). Known concentrations of MF were spiked into the pre‐analysed tablet solution, and the resulting DPV responses were evaluated under identical experimental conditions (0.1 M PBS, pH 5.0, scan rate 50 mV s^−1^) (Table 2). The unspiked tablet sample exhibited a mean MF content of 5.90 µM, consistent with the labelled composition. With successive additions, the measured concentrations increased linearly, yielding nearly 100% recovery. These values fall well within the acceptable analytical range (±10%), confirming the excellent precision and accuracy of the mSPCE‐based method. The narrow variation across replicates highlights the electrode's high reproducibility and minimal matrix interference. Recovery rates above 100% may occur due to matrix effects, surface adsorption/accumulation, calibration deviations, or increased electrode sensitivity [48]. However, compared to previously reported SPCE systems such as NiO/SPCE [49] and CoO/SPCE [50], the current mSPCE exhibits comparable recovery consistency, which can be attributed to its mixed‐valence Cu–Mo–P network that enhances electron mediation and adsorption uniformity. Overall, the results demonstrate the sensor's reliability for quantitative determination of MF in pharmaceutical formulations.

**TABLE 2:** Recovery of MF using mSPCE (*n* = 5, 0.1 M PBS, pH 5.0; scan rate = 50 mV s^−1^).

Sample	Added (µM)	Found (µM, mean ± SD)	Recovery (%)
Tablet	0	5.90 ± 0.08	—
	1.96	7.86 ± 0.12	94.32–107.23
	3.85	9.75 ± 0.15	95.43–105.67
	5.22	11.12 ± 0.18	96.23–104.27

*Note*: The ‘0 µM added’ entry represents the unspiked tablet extract containing the native MF concentration (5.90 ± 0.08 µM, *n* = 5), not a blank solution.

#### 3.3.4 Stability and Reproducibility

The operational stability and reproducibility of the mSPCE sensor were systematically evaluated to ensure its reliability for repeated MF detection. DPV was performed in 0.1 M PBS (pH 5.0) at a scan rate of 50 mV s^−1^ under identical experimental conditions (Figure 7b,d). For the stability assessment, the modified electrode was subjected to seven successive measurements of 1.0 mM MF, yielding highly consistent oxidation peak currents with a relative standard deviation (RSD) of 3.92%. This low RSD value confirms the electrode's excellent operational stability and negligible surface degradation during repeated scans. To examine reproducibility, seven independently prepared mSPCE electrodes were tested under the same conditions (Figure 7c,d). The resulting anodic peak currents exhibited an RSD of 4.82%, indicating outstanding fabrication reproducibility and consistent electrode performance. Such low deviations (<5%) reflect the uniform electrodeposition of the CuMoP composite layer and the structural robustness of the nanostructured coating. These results demonstrate that the mSPCE sensor exhibits excellent stability and reproducibility, comparable to or superior to other reported oxide‐modified SPCE systems. The high repeatability further supports its suitability for routine electrochemical quantification of MF in pharmaceutical formulations and potentially in biological samples [51].

#### 3.3.5 Interference Study

Selectivity is a crucial parameter for assessing the analytical reliability of electrochemical sensors, particularly in complex sample matrices [52]. The interference effects of common inorganic ions and organic compounds on the detection of MF were systematically examined by DPV under optimized conditions (0.1 M PBS, pH 5.0, scan rate 50 mV s^−1^). Various potential interferents, including CO_3_^2−^, SO_4_^2−^, PO_4_^3−^, Ca^2+^, Na^+^ and K^+^, were tested at concentrations 100‐fold higher than those of MF. Additionally, saccharides, including glucose, sucrose, galactose and lactose, were introduced at a 50‐fold concentration; they did not interfere with MF detection at mSPCE. In all cases, the voltammetric response of MF remained nearly unchanged, with current variations of less than 5%, demonstrating the high selectivity of the mSPCE sensor. The absence of significant interference can be attributed to the electrode's mixed‐valence Cu–Mo–P surface, which provides preferential adsorption and rapid charge‐transfer pathways for MF oxidation while minimizing nonspecific interactions with coexisting species. These results confirm that the mSPCE exhibits excellent anti‐interference performance, ensuring accurate and reliable quantification of MF even in the presence of abundant physiological or excipient species.

## 4 Conclusion

This study establishes a scalable and electrochemically controllable strategy for constructing high‐performance ternary CuMoP interfaces on SPCEs for MF sensing. The co‐deposited Cu–Mo–P network forms a mixed‐valence oxide–phosphate architecture that provides a nanostructured, redox‐active interface that amplifies Faradaic responses and stabilizes the electrocatalytic oxidation of MF, surface reactivity and analyte adsorption compared with bare electrodes. Through synergistic redox mediation between Cu^+^/Cu^2+^, electron‐withdrawing Mo^6+^ sites and structurally stabilizing phosphate groups, the modified electrode exhibits strong catalytic amplification and a well‐defined proton‐coupled electron‐transfer pathway for MF oxidation. The resulting sensor delivers high sensitivity, low detection limits (0.85 µM), broad linearity (0.99–13.82 µM) and outstanding selectivity even in the presence of abundant interferents. Real‐sample analysis confirms its quantitative accuracy and suitability for practical pharmaceutical formulations. The excellent stability, reproducibility and recovery (94.32%–107.23%) highlight the robustness of the electrodeposition approach. Overall, this work introduces a versatile, nanostructured multimetal‐phosphate framework that expands the design space for advanced SPCE‐based electrochemical sensors and holds substantial promise for broader pharmaceutical and environmental monitoring applications.

## Author Contributions

Hong Wan contributed to methodology, investigation, validation, visualization, project administration and writing – original draft. Mingfang Zhan contributed to software, data curation, project administration, resources, funding acquisition and writing – review and editing. Chunyan Yin contributed to software, investigation, formal analysis, resources and writing – review and editing. Sima Akter contributed to software, data curation, validation, visualization and writing – review and editing. Sakil Mahmud contributed to conceptualization, formal analysis, supervision and writing – original draft. All authors reviewed and approved the final manuscript.

## Ethics Statement

There are no human/animal subjects in this research, and informed consent is not applicable.

## Disclosure and Declaration of AI Use

Generative AI tools were used solely to enhance language and readability. All content was subsequently reviewed and edited by the authors, who take full responsibility for the final version.

## Conflicts of Interest

The authors declare no conflicts of interest.

## Data Availability

The data that support the findings of this study are available from the corresponding author upon reasonable request.
